# Efficacy and Safety of High-Density Lipoprotein/Apolipoprotein A1 Replacement Therapy in Humans and Mice With Atherosclerosis: A Systematic Review and Meta-Analysis

**DOI:** 10.3389/fcvm.2021.700233

**Published:** 2021-08-02

**Authors:** Ayiguli Abudukeremu, Canxia Huang, Hongwei Li, Runlu Sun, Xiao Liu, Xiaoying Wu, Xiangkun Xie, Jingjing Huang, Jie Zhang, Jinlan Bao, Yuling Zhang

**Affiliations:** ^1^Department of Cardiology, Sun Yat-sen Memorial Hospital, Sun Yat-sen University, Guangzhou, China; ^2^Critical Care Medicine Department, Sun Yat-sen Memorial Hospital, Sun Yat-sen University, Guangzhou, China; ^3^Guangdong Province Key Laboratory of Arrhythmia and Electrophysiology, Sun Yat-sen Memorial Hospital, Sun Yat-sen University, Guangzhou, China; ^4^Comprehensive Department, Sun Yat-sen Memorial Hospital, Sun Yat-sen University, Guangzhou, China

**Keywords:** lipoproteins, HDL, apolipoprotein A-I, mimetic, atherosclerosis

## Abstract

**Background:** Although elevation of HDL-C levels by pharmaceutical drugs have no benefit of cardiovascular endpoint, the effect of high-density lipoprotein/apolipoprotein A1 (HDL/apoA-1) replacement therapy on atherosclerosis is controversial. The current meta-analysis analyzed the effects of HDL/apoA-1 replacement therapies on atherosclerotic lesions both in humans and mice.

**Methods:** The PubMed, Cochrane Library, Web of Science, and EMBASE databases were searched through June 6, 2020. The methodological quality of the human studies was assessed using Review Manager (RevMan, version 5.3.). The methodological quality of the mouse studies was assessed using a stair list. STATA (version 14.0) was used to perform all statistical analyses.

**Results:** Fifteen randomized controlled human trials and 17 animal studies were included. The pooled results showed that HDL/apoA-1 replacement therapy use did not significantly decrease the percent atheroma volume (*p* = 0.766) or total atheroma volume (*p* = 0.510) in acute coronary syndrome (ACS) patients (*N* = 754). However, HDL/apoA-1 replacement therapies were significantly associated with the final percent lesion area, final lesion area, and changes in lesion area (SMD, −1.75; 95% CI: −2.21~-1.29, *p* = 0.000; SMD, −0.78; 95% CI: −1.18~-0.38, *p* = 0.000; SMD: −2.06; 95% CI, −3.92~-0.2, *p* = 0.03, respectively) in mice.

**Conclusions:** HDL/apoA-1 replacement therapies are safe but do not significantly improve arterial atheroma volume in humans. The results in animals suggest that HDL/apoA-1 replacement therapies decrease the lesion area. Additional studies are needed to investigate and explain the differences in HDL/apoA-1 replacement therapy efficacies between humans and animals.

**Trial registration number:** Human pooled analysis: PROSPERO, CRD42020210772. prospectively registered.

## Key Points

1 HDL/apoA-1 replacement therapies significantly benefit plaque inhibition in atherosclerosis mouse models but do not significantly benefit human atheroma volume.
2 Trials with mice preliminarily suggest that HDL/apoA-1 replacement therapies more effectively inhibit atherosclerosis development in earlier stages than in mature lesions.

## Introduction

Cardiovascular disease (CVD) claimed an estimated 17.9 million lives in 2016, and it remains the leading cause of death worldwide, representing 31% of all global deaths (1). Effective strategies for preventing and treating CVD is a major public health challenge worldwide. Lipid modification is the major method for cardiovascular risk reduction, especially low-density lipoprotein cholesterol (LDL-C)-lowering therapy, but patients with controlled LDL-C in the normal range still have increased residual cardiovascular risk (2). High-density lipoprotein (HDL) plays a crucial role in cholesterol reverse transportation and metabolism. Since Miller discovered that a reduction in plasma HDL concentrations may accelerate the development of atherosclerosis, HDL-C has become a new focus in the investigation of preventive therapy for CVD (3). However, studies over the past several decades that increased the self-production of HDL-C showed different results regarding CVD prevention (4–8). A great number of studies have been performed to explain these differences. (1) HDL is a conglomerate of protein, triglycerides, phospholipids, cholesterol esters, and cholesterol that performs various functions, including cholesterol efflux and reverse cholesterol transportation, anti-inflammatory effects, antioxidant effects, nitric oxide-promoting and endothelial function-enhancing effects, antithrombotic effects, and antiapoptotic effects (9). (2) HDL particles that fail to perform the biological functions mentioned above are termed “dysfunctional.” (3) Changes in HDL composition may cause dysfunction. Serum amyloid A is a significant predictor of cardiovascular disease risk, and it replaces apoA-1 in HDL during acute phase induction and prevents access of HDL to the plasma membrane (10, 11). Reductions in the antioxidative and anti-inflammatory properties of HDL-associated enzymes, such as paraoxonase 1 activity, lecithin cholesterol acyltransferase deficiency were accompanied by HDL oxidation and the promotion of atherosclerotic lesion formation (12, 13). Changes in lipid components, including triglyceride(s) and cholesteryl ester, also caused HDL dysfunction (14). In addition to losing its cardioprotective role, dysfunctional HDL may be harmful to patients *via* conversion into a proinflammatory and pro-oxidant component that promotes LDL oxidation (15, 16). Therefore, the use of normal-functioning HDL/apoA-1 or mimetics may ultimately elucidate the role of HDL/apoA-1 in CVD prevention.

Thus, in the present meta-analysis, we aimed to investigate the efficiency of HDL/apoA-1 replacement therapies in mouse models and patients with atherosclerosis.

## Methods

The meta-analysis was performed according to the Preferred Reporting Items for Systematic Reviews and Meta-Analyses (PRISMA) statement (Supplementary Table 1). The methodological quality of the human studies was assessed using Review Manager (RevMan) software (version 5.3). The methodological quality of the mouse studies was assessed by using a stair list.

Participants included in the efficacy analysis were acute coronary syndrome (ACS) patients. The intervention was HDL/apoA-1 replacement therapy. The comparison was atheroma volume in the coronary artery measured via intravascular ultrasonography. The outcomes were changes in percent atheroma volume (PAV) and total atheroma volume (TAV), and the study type was randomized controlled trials. The mice included in the efficacy analysis were apolipoprotein E-deficient or low-density lipoprotein receptor-deficient mice fed with regular rodent chow or a high-fat diet. The intervention was HDL/apo-A1 replacement therapy. Comparisons included the lesion areas in the aortic sinus, aortic root, aorta, innominate artery, or carotid arteries. The outcomes were the final percent lesion area, final lesion area, and changes in lesion area. The study type was limited to animal-controlled studies.

### Search Strategy

Two authors (AA and CH) searched the PubMed, Cochrane Library, Web of Science, and EMBASE databases up to June 6, 2020, for eligible studies using wide search terms and included all publications that met the inclusion criteria (Supplementary Table 2). We used the following terms: “Lipoproteins, HDL” or “Apolipoprotein A-I” and “mimetic” or “recombinant HDL” and “coronary artery disease” or “Cardiovascular Diseases” for the literature search. The reference lists from relevant articles were reviewed to identify additional studies.

### Inclusion Criteria and Study Selection

Studies meeting the following criteria were included: (1) studies reporting the effect or safety of HDL/apoA-1 replacement therapies on atherosclerosis in mouse models and patients with atherosclerosis, (2) human studies designed as randomized controlled trials (RCTs), and (3) studies written in English. Studies with insufficient data or gray literature were excluded.

### Data Extraction

Two independent reviewers (AA and CH) screened the titles and abstracts for relevance. Disagreements between reviewers were discussed until a consensus was reached. The manuscripts of selected titles/abstracts were assessed for inclusion. Using the selection criteria listed above, the two reviewers independently extracted baseline information using predefined extraction flow sheets. We resolved any disagreements between the authors via discussion. A third review author (HL) arbitrated when differences in opinions emerged. If a study compared different HDL/apoA-1 replacement therapies with a replacement therapy-naïve cohort, the data of all replacement therapies were separately compared with the control group.

### Data Analysis

Statistical analyses were performed using Stata (version 14.0). A random-effects model with I-V heterogeneity (continuous data) or the Mantel–Haenszel (binary data) method was used to calculate pooled standardized mean differences (SMDs), or odds ratios (ORs) and 95% confidence intervals (CIs). We assessed several outcomes in human trials: PAV, TAV in the coronary artery, and other outcomes representing safety. We assessed three outcomes in animal trials: final percent lesion area, final lesion area, and changes in lesion area in arteries. Heterogeneity between studies was assessed using the *Q*-test and I^2^ statistic. Heterogeneity was considered significant when the *p*-value was < 0.05. Publication bias was assessed using visual inspection of funnel plots and Egger's regression test. The influence of individual studies was examined by the removal of one study at a time.

## Results

### Human Trials

#### Study Outlines and Characteristics

**Figure 1:**
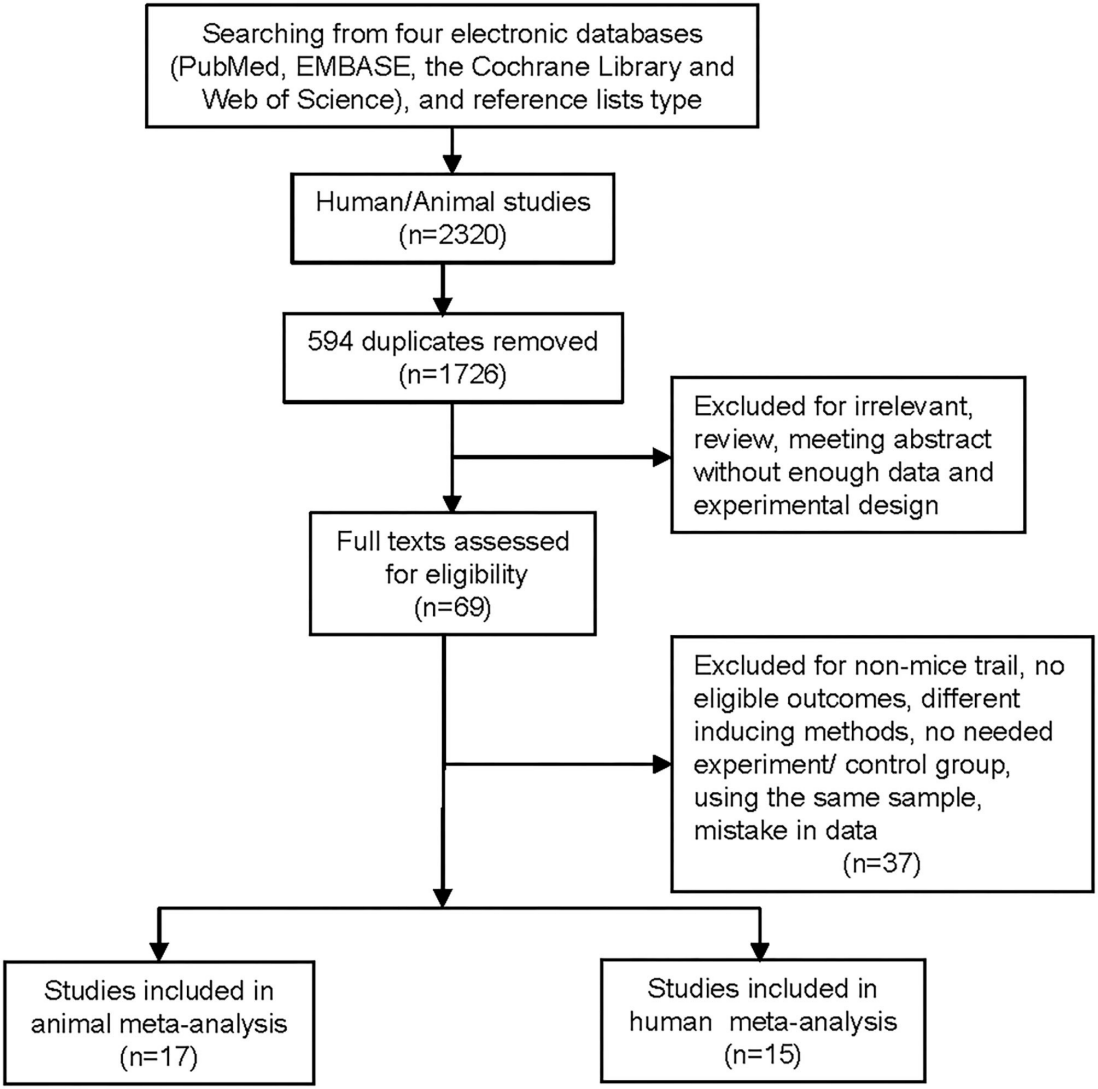
Flowchart of the literature search and study selection process.

We identified 15 RCTs, of which 6 trials, including 754 ACS patients (replacement therapies = 414, placebo = 340), were used for efficacy analysis, and all 15 trials were used for safety analysis (Table 1) (17–31). A flowchart of the selection of eligible trails is shown in Figure 1. The duration of replacement therapy administration in the included studies ranged from a single administration to 10 weeks (weekly administration). The risk of bias of the included studies is shown in the Supplementary Figure 1.

**Table 1:** Characteristics of the studies included in the human meta-analysis.

**References**	**Country**	**Sample size**	**Intervention and dose**	**Duration**	**Participants**	**Mean age (years)**	**Mean BMI**	**Diabetes %**	**HDL-C (mg/dL)**	**ApoA-1 (mg/dL)**	**LDL-C (mg/dL)**
Nicholls et al. (17)	Multi-stage	301	Placebo vs. CER-001 (3 mg/kg)	Weekly/10 weeks	ACS	59.84	29.15	19.49	40	127.01	83.48
Tardif et al. (18)	Multi-stage	507	Placebo vs. CER-0010 (3/6/12 mg/kg)	Weekly/6 weeks	ACS	58.98	NA	24.89	NA	131.42	NA
Nicholls et al. (19)	Multi-stage	126	Placebo vs. MDCO-216 (20 mg/kg)	Weekly/5 weeks	ACS	61.78	28.48	20	43.4	123.15	88.67
Kallend et al. (20)	Netherlands	48	Placebo vs. MDCO-216 (HV: 5/10/20/30/40 mg/kg) (CAD: 10/20/30/40 mg/kg)	Single infusion	Stable CHD, healthy volunteers	HV:25.6 CAD:62.2	HV:22.5 CAD:27	NA	HV:55.3 CAD:46.79	HV:157.3 CAD:121.5	HV:84.3 CAD:85.85
Gibson et al. (31)	Multi-stage	1,258	Placebo vs. CSL112 (2/6 g apoA-I per dose)	Weekly/4 weeks	AMI	58.33	28.85	22.26	40.87	126.14	92.97
Gibson et al. (21)	Multi-stage	83	Placebo vs. CSL112(6 g)	Weekly/4 weeks	AMI	71.04	29.49	42.17	42.42	114.08	83.78
Tricoci et al. (22)	United States	45	Placebo vs. CSL112 (1.7/3.4/6.8 g)	Single infusion	Stable atherosclerosis disease	59	30	25	NA	NA	NA
Easton et al. (23)	Australia	36	Placebo vs. CSL112 (3.4/6.8 g/3.4 g)	Weekly/weekly/twice weekly (4 weeks)	Healthy subjects	25.25	25.05	NA	NA	122.5	NA
Tortorici et al. (24) (NRF, MRI)	Germany, United Kingdom	32	Placebo vs. CSL112 (2/6 g)	Single infusion	Healthy subjects	NRF:55.63 MRI:69.13	NA	NA	NA	NA	NA
Tardif et al. (25)	Canada	183	Placebo vs. CSL111 (40/80 mg/kg)	Weekly/4 weeks	ACS	57.7	NA	NA	NA	NA	NA
Nissen et al. (26)	USA	57	Placebo vs. ETC-216 (15/45 mg/kg)	Weekly/5 weeks	ACS	57.27	NA	20.22	42.1	NA	81.9
Waksman et al. (27)	USA	28	Placebo vs. LS PDS-2 Devise Delipidation	Weekly/7 weeks	ACS	55	NA	14.04	41.16	NA	119.05
Dunbar et al. (28)	Pennsylvania	62	Placebo vs. D-4F (100/300/500 mg)	Daily/13 days	Stable CHD or equivalent risk	60.5	31.7	28.57	44.46	133.24	76.38
Watson et al. (29) (IV)	Multi-stage	72	Placebo vs. L-4F (3/10/30/100 mg)	Daily/7 days	Stable CHD or a CHD equivalent	60.16	29.54	54.84	46.34	116.54	81.8
Watson et al. (29) (SC)	Multi-stage	104	Placebo vs. L-4F (10/30 mg)	Daily/28 days	Stable CHD or a CHD equivalent	60.43	29.55	59.72	44.51	128.64	NA
Bloedon et al. (30)	Pennsylvania	50	Placebo vs. D-4F (30/100/300/500 mg)	Single oral administration	Stable CHD or a CHD equivalent	59.9	31.3	75.93	42.86	122.76	NA

*HV, healthy volunteer; CAD, coronary artery disease; CHD, coronary heart disease; ACS, acute coronary syndrome; AMI, acute myocardial infarction; ASD, stable atherosclerotic disease; NA, not available; HDL-C, high-density lipoprotein; apoA-1, apolipoprotein A-1; LDL-C, low-density lipoprotein; CHDq, coronary heart disease equivalent*.

#### Percent Atheroma Volume and Total Atheroma Volume

**Figure 2:**
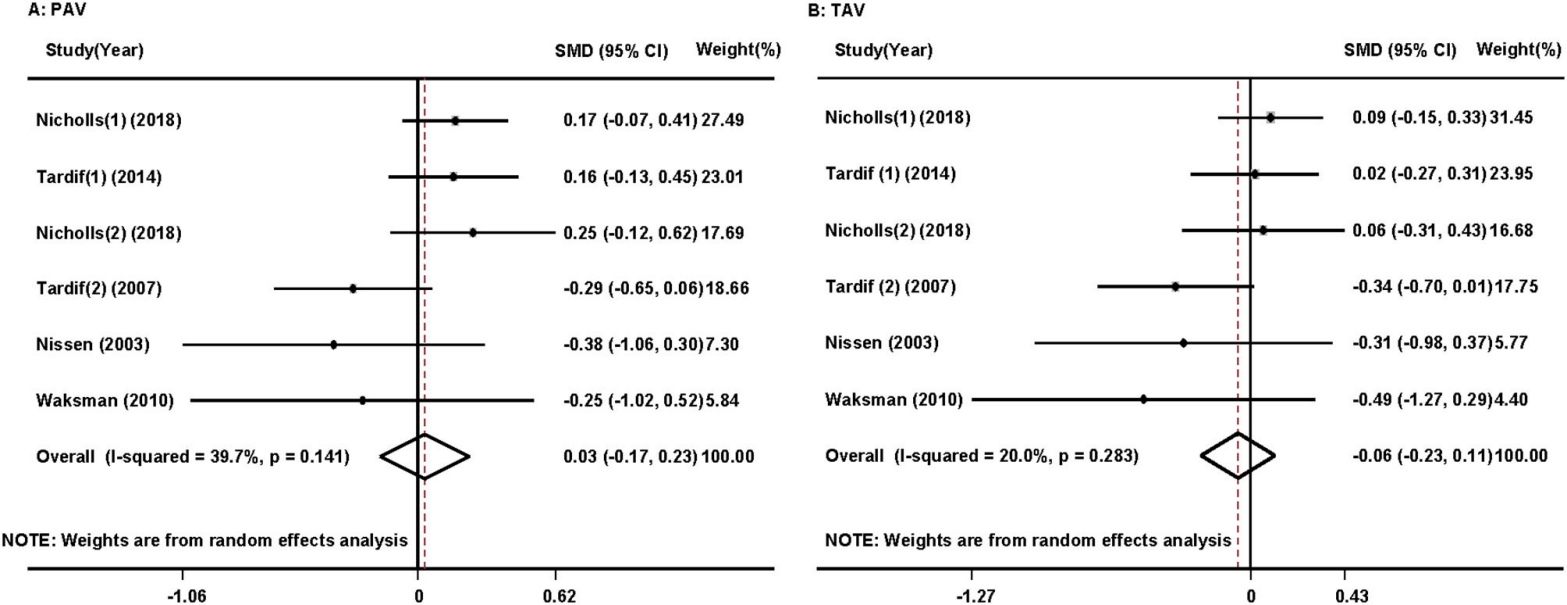
Forest plots of the meta-analysis of the associations between percent atheroma volume (PAV) **(A)** and total atheroma volume (TAV) **(B)** in the coronary artery and high-density lipoprotein/apolipoprotein A1 (HDL/apoA-1) replacement therapy administration in acute coronary syndrome patients using a random-effects model. SMD, standardized mean difference; CI, confidence interval. This result shows that HDL/apoA-1 replacement therapies did not significantly decrease PAV or TAV in patients with acute coronary syndrome.

Pooled analysis revealed that HDL/apoA-1 replacement therapies did not significantly decrease PAV (SMD:0.03; 95% CI −0.17~0.23, *p* = 0.766, I^2^ = 39.7%) or TAV (SMD: −0.06; 95% CI −0.23~0.11, *p* = 0.510, I^2^ = 20.0%) in patients with atherosclerosis (Figure 2) (17–19, 25–27). The results did not significantly change in the sensitivity analysis (Supplementary Figures 2A,B).

#### Safety Assessment of High-Density Lipoprotein/Apolipoprotein A1 Replacement Therapies

Several studies separately reported the adverse effects of HDL/apoA-1 replacement therapies, including headache (*n* = 8), renal impairment (*n* = 6), hepatic impairment (*n* = 11), and nausea, vomiting, and abdominal pain (*n* = 7). Pooled analysis revealed that HDL/apoA-1 replacement therapies were safe, with no significantly increased risk of adverse events (headache: OR 1.58, 95% CI 0.84–2.97, I^2^ = 0.0%; renal impairment: OR 0.76, 95% CI 0.29–1.99, I^2^ = 8.7%; hepatic impairment: OR 1.37, 95% CI 0.51–3.64, I^2^ = 34.9%; nausea, vomiting, or abdominal pain: OR 0.81; 95% CI 0.47–1.38, I^2^ = 0.0%) (Supplementary Figure 3). The results did not significantly change in the sensitivity analysis (Supplementary Figure 4).

### Mouse Trials

#### Study Outlines and Characteristics

We identified 17 controlled trials that included 479 atherosclerotic mice (HDL/apoA-1 replacement therapies = 285, placebo = 194) (Supplementary Table 3) (32–45). A flowchart of the selection of eligible trails is shown in Figure 1. The therapy administration duration ranged from 30 days to 16 weeks. The scientific inquiry of the animal trials was assessed using a stair list that included 12 “yes,” 95 “no,” and 5 “unclear” (Supplementary Table 5). Funnel plots were provided in Supplementary Figures 6A,B.

#### Final Percent Lesion Area

**Figure 3:**
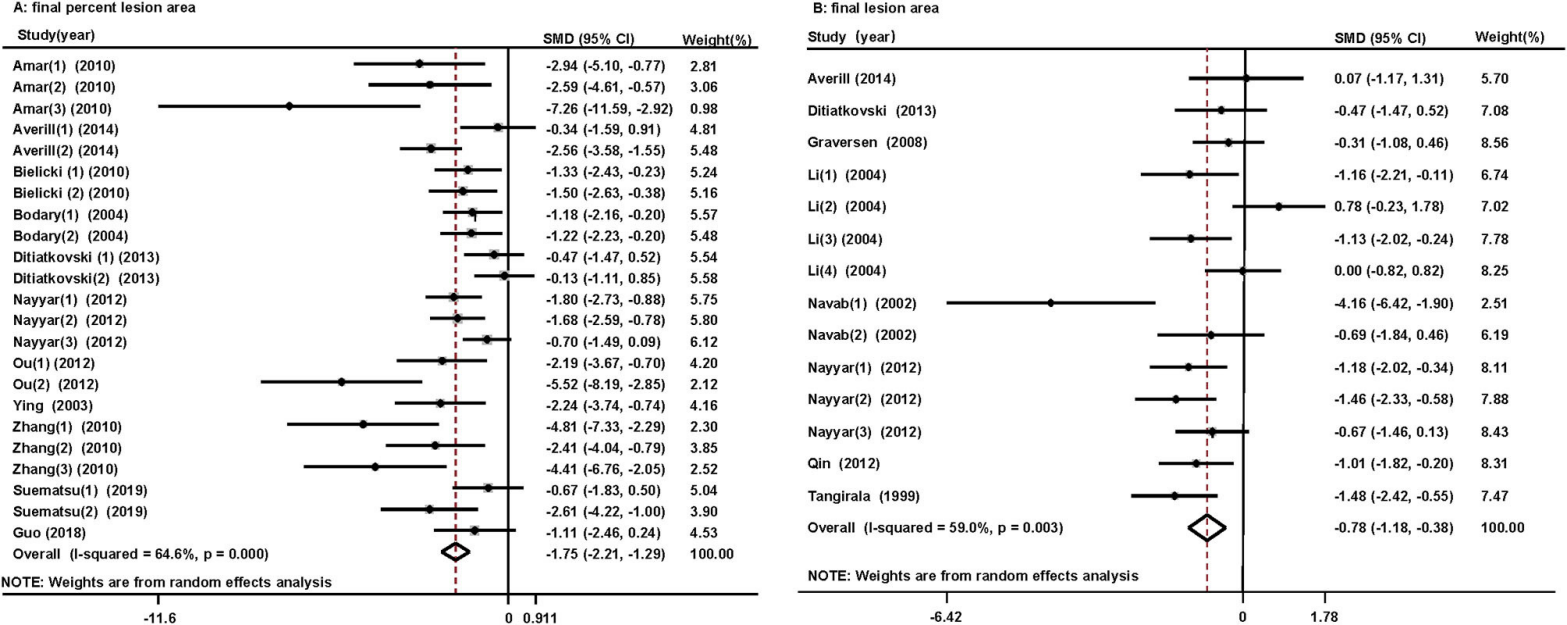
Forest plots of the meta-analysis of the associations between final percent lesion area **(A)**, final lesion area **(B)** in arteries, and HDL/apoA-1 replacement therapy administration in mice using a random-effects model. SMD, standardized mean difference; CI, confidence interval. This result shows that HDL/apoA-1 replacement therapies significantly decreased the final percent lesion area and final lesion area in mice with coronary atherosclerosis.

Eleven studies reported the effects of HDL/apoA-1 replacement therapies on the percent lesion area in mice (32–37, 40, 41, 46–48). A reduced final percent lesion area was observed in the HDL/apoA-1 replacement therapy-treated groups (SMD, −1.75; 95% CI: −2.21~-1.29, I^2^ = 64.6%) (Figure 3A). The results did not significantly change in the sensitivity analysis (Supplementary Figure 6C).

#### Final Lesion Area

Eight studies reported the effect of HDL/apoA-1 replacement therapies on the final lesion area in mice (33, 36, 38–40, 42, 43, 45). A reduced final lesion area was observed in the HDL/apoA-1 replacement therapy group (SMD, −0.78; 95% CI: −1.18~-0.38, I^2^ = 59%) (Figure 3B). The results did not significantly change in the sensitivity analysis (Supplementary Figure 6D).

#### Changes in Lesion Area

Three studies reported the effects of HDL/apoA-1 replacement therapies on changes in lesion area in mice (37, 43, 44). More changes in lesion area (reduction) were observed in the HDL/apoA-1 group than the control group (SMD: −2.06; 95% CI, −3.92~-0.2, I^2^ = 83.9%) (Supplementary Figure 5).

#### Subgroup Analysis

A reduced final percent lesion area was observed in HDL/apoA-1 subgroups based on LDL^−/−^ mice, apoE^−/−^ mice, males, and females. A reduced final lesion area was observed in HDL/apoA-1 subgroups based on females and apoE^−/−^ mice (Supplementary Table 4).

## Discussion

To our knowledge, this study is the first meta-analysis of the effects of HDL/apoA-1 replacement therapies on atherosclerotic lesions. The pooled estimate in animal studies showed that HDL/apoA-1 replacement therapies were associated with a significant reduction in the final percent lesion area and final lesion area and a substantial decrease in the lesion area. However, we failed to find a significant association between HDL/apoA-1 replacement therapies and PAV or TAV in the pooled human results.

HDL plays several crucial roles in atherosclerotic prevention, including cholesterol efflux capacity, antioxidant activity, anti-inflammatory activity, cytoprotective activity, and vasodilatory activity (49). However, some abnormal conditions, including inflammatory conditions and metabolic diseases, cause HDL dysfunction by affecting its components, as described in the *Introduction* section (50–52). This change in HDL may underlie the failure to prevent atherosclerosis by increasing HDL production without considering its dysfunction. For example, increasing HDL by inhibiting cholesteryl ester transfer protein (CETP) did not reduce the rate of cardiovascular events despite inducing a 133.2% increase in HDL-C levels (53). One case reported a 52-year-old white female with an extremely high HDL level of 218 mg/dl who suffered from coronary artery disease (54). Therefore, improving HDL dysfunction may be more beneficial for atherosclerosis prevention than increasing its level.

Previously published animal studies suggest that HDL/apoA-1 replacement therapies (considered equal to normal-functioning HDL/apoA-1) have a significant beneficial effect on atherosclerosis prevention. Our pooled animal trials also revealed that HDL/apoA-1 replacement therapies had a significant preventive effect on the lesion area, which is consistent with our hypothesis that providing normal-functioning HDL/apoA-1 would be effective for atherosclerosis prevention. This strategy should also be effective in atherosclerosis prevention in humans. However, the cause of the failure of HDL/apoA-1 replacement therapies to provide beneficial effects on atherosclerosis in humans is not known. We summarized the possible reasons below.

1. HDL/apoA-1 replacement therapies may be more effective in preventing early-stage atherosclerotic lesions than altering mature lesions, which was demonstrated in animal trials (38, 44) because atherosclerosis is a chronic inflammatory process. Mohanta et al. proposed a new concept that the disruption of the balance between pro- and antiatherogenic immune cell subsets due to the exhaustion of anti-inflammatory and immune-suppressing cells during sustained inflammatory processes may trigger clinically overt atherosclerosis (55). Reconstituted discoidal HDL and apoA-1 inhibit inflammation in endothelial cells (56, 57). Lipid-free apoA-1 also inhibits macrophage activation via three main pathways (58–60). These studies suggest that earlier administration of HDL/apoA-1 replacement therapy would prevent the sustained inflammatory process that triggers a clinically overt atherosclerosis process, which is consistent with the discovery of Li et al. that D4F decreased the intraplaque lipid and macrophage content in evolving atherosclerosis (38). However, the balance between pro- and antiatherogenic immune cell subsets is already damaged in mature atherosclerotic lesions, which weakens the atheroprotective role of HDL/apoA-1 replacement therapy.

2. Does the administered HDL/apoA-1 replacement therapy maintain normal functioning in participants with a dysfunctional internal environment or after conversion into pro-inflammatory and pro-oxidant environments? The apoA-1 mimetic peptide D4F caused pre-β HDL formation, improved HDL-mediated cholesterol efflux, and reversed cholesterol transportation from macrophages in mice and rendered HDL less inflammatory in high-risk coronary heart disease patients (28, 61). However, two distinct clinical studies that included patients with coronary heart disease tested the impact of the apoA-1 mimetic peptide L-4F and failed to demonstrate its role in the HDL-inflammatory index and paraoxonase activity improvement, but it increased the high-sensitivity C-reactive protein levels, which was paradoxical to an *ex vivo* study (29). Therefore, the functional transformation of HDL/apoA-1 replacement therapy after administration to the body should be further analyzed to guide the prevention of atherosclerosis by increasing the “good” HDL.

3. Differences in lipid metabolism-relevant gene expression between humans and mice, including cholesteryl ester transfer protein, which is absent from the mouse genome but provides an avenue for the delivery of cholesterol from HDL to LDL in humans, may also be responsible for the different outcomes both *in vivo* and *in vitro* (mentioned above) after HDL/apoA-1 replacement therapy administration (62). Therefore, further human-specific analyses must be performed.

4. The duration and frequency of HDL/apoA-1 replacement therapy administration may also explain the different results in human and mouse trials (Table 1 and Supplementary Table 3). The human lifespan is much longer than that of mice, and the process of lesion development in humans is likely much more complicated. However, the duration and frequency of replacement therapy administration in some human studies were shorter than those in mouse studies. Therefore, the frequency and doses at the beginning of the replacement therapy administration period should be further investigated.

Further RCTs should investigate the preventive role of HDL/apoA-1 replacement therapies on atherosclerosis development. The participants would be healthy volunteers with atherosclerotic lesions in the early stage, and the duration and dose of the replacement therapy would be long and adequate.

However, before comparing the role of HDL/apoA-1 replacement therapy on atherosclerotic plaques between mice and humans, we must consider atherosclerosis progression in apoE^−/−^ and LDLR^−/−^ mice accompanied by expansive remodeling, which causes the absence of luminal narrowing that is different from humans, especially in ACS patients (63). However, mouse models have shown that apo A-I overexpression consistently reduces atherosclerosis progression independent of the genetic and metabolic context by increasing HDL cholesterol, which suggests their partial comparability (64). Therefore, animal trials may be a “lighthouse” for clinical trials, but we should not overestimate their guiding function.

## Limitation

Our pooled analysis has several limitations. The methods used to evaluate vascular atherosclerosis were different in humans and mice (human: atheroma volume, mice: lesion area) because expansive remodeling of the vasculature accompanying atherosclerosis progression in mice caused the absence of luminal narrowing (63). The small number of studies in the human pooled analyses of efficacy is a limitation, and further investigations with earlier and longer administration times and increased doses should be performed. Comparisons of functional tests, including anti-inflammatory, paraoxonase activity, and RCTs, between humans and mice are absent because of data restrictions.

## Conclusion

Evidence from a pooled analysis suggests that HDL/apoA-1 replacement therapies reduce the atherosclerotic lesion area in mice but do not show a beneficial effect on human atheroma volume despite being considered safe. Additional studies are needed to further investigate and explain the different effects of HDL/apoA1 replacement therapies in humans and mice.

## Data Availability Statement

The original contributions presented in the study are included in the article/Supplementary Material, further inquiries can be directed to the corresponding author/s.

## Author Contributions

AA and CH: searched for articles, filtered the included articles, performed statistical analyses, and drafted the manuscript. HL: helped establish the inclusion and exclusion criteria, settled conflicts in literature inclusion, and evaluated the included studies. RS: performed statistical analyses and created the tables and figures. XL and XW: evaluated the included studies and performed the statistical analyses. XX and JH: searched for articles and selected included articles. JZ and JB: helped generate the figures and tables. YZ: designed the topic of the review, established the inclusion and exclusion criteria, and revised the manuscript. All authors read and approved the final manuscript.

## Conflict of Interest

The authors declare that the research was conducted in the absence of any commercial or financial relationships that could be construed as a potential conflict of interest.

## Publisher's Note

All claims expressed in this article are solely those of the authors and do not necessarily represent those of their affiliated organizations, or those of the publisher, the editors and the reviewers. Any product that may be evaluated in this article, or claim that may be made by its manufacturer, is not guaranteed or endorsed by the publisher.
