# Supplementary material for: Efficacy and Safety of High-Density Lipoprotein/Apolipoprotein A1 Replacement Therapy in Humans and Mice With Atherosclerosis: A Systematic Review and Meta-Analysis
Source: Front Cardiovasc Med. 2021 Aug 2;8:700233. doi: 10.3389/fcvm.2021.700233 (PMC8377725; doi:10.3389/fcvm.2021.700233)
Supplement: Supplementary file 1 [file Data_Sheet_1.docx]

Efficacy and safety of HDL/apoA-1 replacement therapy in humans and mice with atherosclerosis: A systematic review and meta-analysis

Ayiguli Abudukeremu^1,#^, Canxia Huang^3,#^,Hongwei Li^1^, Runlu Sun^1,4^, Xiao Liu^1,4^, Xiaoying Wu^1,4^, Xiangkun Xie^1,4^, Jingjing Huang^1,4^, Jie Zhang^1,4^, Jinlan Bao^2^, Yuling Zhang^1,4,*^

^1^Cardiovascular Medicine Department, Sun Yat-sen Memorial Hospital, Sun Yat-sen University, No. 107, the West of Yanjiang Road West, Guangzhou, 510120, China

^2^Comprehensive Department, Sun Yat-sen Memorial Hospital, Sun Yat-sen University, No. 107, the West of Yanjiang Road West, Guangzhou, 510120, China

^3^Critical Care Medicine Department, Sun Yat-sen Memorial Hospital, Sun Yat-sen University, No. 107, the West of Yanjiang Road West, Guangzhou, 510120, China

^4^Guangdong Province Key Laboratory of Arrhythmia and Electrophysiology, Guangzhou, 510120, China

^#^Ayiguli Abudukeremu and Canxia Huang have equal contributions.

*Correspondence: Yuling Zhang, Department of Cardiology, Sun Yat-sen Memorial

Hospital, Sun Yat-sen University No. 107, the West of Yanjiang Road, Yuexiu District Guangzhou, China, 510120, Email: [zhyul@mail.sysu.edu.cn](mailto:zhyul@mail.sysu.edu.cn).

| **Section/topic** | **#** | **Checklist item** | **Reported on page #** |
| --- | --- | --- | --- |
| **TITLE** | | |  |
| Title | 1 | Identify the report as a systematic review, meta-analysis, or both. | 1 |
| **ABSTRACT** | | |  |
| Structured summary | 2 | Provide a structured summary including, as applicable: background; objectives; data sources; study eligibility criteria, participants, and interventions; study appraisal and synthesis methods; results; limitations; conclusions and implications of key findings; systematic review registration number. | 2 |
| **INTRODUCTION** | | |  |
| Rationale | 3 | Describe the rationale for the review in the context of what is already known. | 3 |
| Objectives | 3 | Provide an explicit statement of questions being addressed with reference to participants, interventions, comparisons, outcomes, and study design (PICOS). | 4 |
| **METHODS** | | |  |
| Protocol and registration | 3 | Indicate if a review protocol exists, if and where it can be accessed (e.g., Web address), and, if available, provide registration information including registration number. | 2 |
| Eligibility criteria | 4 | Specify study characteristics (e.g., PICOS, length of follow-up) and report characteristics (e.g., years considered, language, publication status) used as criteria for eligibility, giving rationale. | 5 |
| Information sources | 4 | Describe all information sources (e.g., databases with dates of coverage, contact with study authors to identify additional studies) in the search and date last searched. | 5 |
| Search | 4 | Present full electronic search strategy for at least one database, including any limits used, such that it could be repeated. | supplementary |
| Study selection | 4 | State the process for selecting studies (i.e., screening, eligibility, included in systematic review, and, if applicable, included in the meta-analysis). | 5 |
| Data collection process | 4 | Describe method of data extraction from reports (e.g., piloted forms, independently, in duplicate) and any processes for obtaining and confirming data from investigators. | 5 |
| Data items | 5 | List and define all variables for which data were sought (e.g., PICOS, funding sources) and any assumptions and simplifications made. | 5 |
| Risk of bias in individual studies | 5 | Describe methods used for assessing risk of bias of individual studies (including specification of whether this was done at the study or outcome level), and how this information is to be used in any data synthesis. | 2 |
| Summary measures | 4-5 | State the principal summary measures (e.g., risk ratio, difference in means). | 5-6 |
| Synthesis of results | 4-5 | Describe the methods of handling data and combining results of studies, if done, including measures of consistency (e.g., I^2^) for each meta-analysis. | 5-6 |

**Supplementary table1: PRISMA statement list.** We used PRISMA statement list for reporting Items

**Supplementary Table 2: EMBASE search strategy.**

| 1 | 'cardiovascular disease'/exp |
| --- | --- |
| 2 | 'cardiovascular diseases':ab,ti OR 'cardiovascular disease':ab,ti OR 'disease, cardiovascular':ab,ti OR 'diseases, cardiovascular':ab,ti |
| 3 | 1 OR 2 |
| 4 | 'high density lipoprotein'/exp |
| 5 | 'lipoproteins,hdl':ab,ti OR 'hdl lipoproteins':ab,ti OR 'heavy lipoproteins':ab,ti OR 'lipoproteins, heavy':ab,ti OR 'high-density lipoproteins':ab,ti OR 'high density lipoproteins':ab,ti OR 'lipoproteins, high-density':ab,ti OR 'alpha lipoproteins':ab,ti OR 'alpha-1 lipoprotein':ab,ti |
| 6 | 4 OR 5 |
| 7 | 'apolipoprotein a1'/exp |
| 8 | 'apolipoprotein a-i':ab,ti OR 'apolipoprotein a i':ab,ti OR 'apo a-i':ab,ti OR 'apo a1':ab,ti OR 'apolipoprotein ai':ab,ti OR 'apoa 1':ab,ti OR 'apoa i':ab,ti OR 'apolipoprotein a-1':ab,ti OR 'apolipoprotein a 1':ab,ti OR 'apolipoprotein a1':ab,ti OR 'apo a-1':ab,ti OR 'apo ai':ab,ti OR 'pro-apolipoprotein a-i':ab,ti OR 'pro apolipoprotein a i':ab,ti OR 'pro-apo a-i':ab,ti OR 'pro apo a i':ab,ti OR 'proapolipoprotein ai':ab,ti OR 'apolipoprotein ai propeptide':ab,ti OR 'apolipoprotein a-i isoproteins':ab,ti OR 'apolipoprotein a i isoproteins':ab,ti OR 'apo a-i isoproteins':ab,ti OR 'apo a i isoproteins':ab,ti OR 'apolipoprotein a-i isoprotein-2':ab,ti OR 'apolipoprotein a i isoprotein 2':ab,ti OR 'apolipoprotein a-i isoprotein-4':ab,ti OR 'apolipoprotein a i isoprotein 4':ab,ti |
| 9 | 7 OR 8 |
| 10 | 6 OR 9 |
| 11 | 3 AND 10 |
| 12 | 'mimetic peptide*':ab,ti OR nanoparticle*:ab,ti OR 'artificial hdl':ab,ti OR 'reconstituded hdl':ab,ti OR 'recombinant hdl':ab,ti OR mimetic*:ab,ti |
| 13 | 11 AND 12 |

We searched PubMed, Cochrane, Web of Science, EMBASE databases up to June 6, 2020 for eligible studies using wide search terms, and present EMBASE search strategy as example.

| study  **Supplementary Table3. Characteristics of Studies Included in the mice Meta-Analysis** | year | Animal | diet | Gender | N | Intervention | Route of administration | Dose | Duration | The artery used for lesion assessment |
| --- | --- | --- | --- | --- | --- | --- | --- | --- | --- | --- |
| Amar (1) | 2010 | apoE -/- mice | regular rodent chow diet | female | 8 | 5A peptide | iv | 30 mg/kg 5A | Three times a week/13weeks | aorta |
| Amar (2) | 2010 | apoE -/- mice | regular rodent chow diet | female | 8 | 5A peptide | iv | 30 mg/kg 5A | Three times a week/13weeks | aorta |
| Amar (3) | 2010 | apoE -/- mice | regular rodent chow diet | female | 8 | 5A peptide | ip | 30 mg/kg 5A | Three times a week/13weeks | aorta |
| Averill (1) | 2014 | LDLR -/- mice | high fat high sucrose with cholesterol | male | 10 | L4F mimetic peptide | ih | 100 mg/ mouse | Daily/8 weeks | The brachiocephalic artery |
| Averill (2) | 2014 | LDLR -/- mice | high fat high sucrose with cholesterol | male | 10 | apoA-I overexpression | ih | 100 mg/ mouse | Daily/8 weeks | The brachiocephalic artery |
| Bielicki (1) | 2010 | apoE -/- mice | high-fat Western diet | male | 16 | ATI-5261 | ip | 30 mg/kg | Alternate days/  6 weeks | from the middle of the ventricle to aorta; descending thoracic- and abdominal-aorta； aortic sinus plaque |
| Bielicki (2) | 2010 | LDLR -/- mice | high-fat Western diet | male | 16 | ATI-5261 | ip | 30 mg/kg | Daily/6 weeks | from the middle of the ventricle to aorta; descending thoracic- and abdominal-aorta； aortic sinus plaque |
| Bodary (1) | 2004 | LDLR -/- mice | western chow diet | male | 19 | ESP 24218 | Gene transfection | / | / | aortic arch. |
| Bodary (2) | 2004 | LDLR -/- mice | western chow diet | male | 18 | wild-type ApoA-I | Gene transfection | / | / | aortic arch. |
| Ditiatkovski (1) | 2013 | apoE -/- mice | high fat diet | male | 16 | ELK-2A2K2E | ip | 30 mg/kg | Three times per week/16 weeks | Aortae |
| study | year | Animal | diet | Gender | N | Intervention | Route of administration | Dose | Duration | The artery used for lesion assessment |
| Ditiatkovski (2) | 2013 | apoE -/- mice | high fat diet | male | 16 | ELK-2A2K2E | ip | 30 mg/kg | Three times per week/16 weeks | Aortae |
| Guo | 2018 | apoE -/- mice | atherogenic diet | male | 10 | ApoA-I mimetic peptides 22A | ip | 30 mg/kg | Three times a week/6 weeks | aortic root （aortic sinus ） |
| Li (1) | 2004 | apoE -/- mice | high-fat, high-cholesterol diet | Not mentioned | 17 | D4F | ip | 50 μg | Daily/4 weeks | vein-graft atherosclerotic |
| Li (2) | 2004 | apoE -/- mice | high-fat, high-cholesterol diet | Not mentioned | 17 | D4F | ip | 50 μg | Daily/4 weeks | aortic sinus |
| Li (3) | 2004 | apoE -/- mice | high-fat, high-cholesterol diet | Not mentioned | 17 | D4F | ip | 50 μg | Daily/4 weeks | vein-graft atherosclerotic |
| Li (4) | 2004 | apoE -/- mice | high-fat, high-cholesterol diet | Not mentioned | 17 | D4F | ip | 50 μg | Daily/4 weeks | aortic sinus |
| Navab (1) | 2002 | LDLR -/- mice | Western diet | female | 11 | D-4F | oral | 2.5mg | Twice daily/  6 weeks | aortic root |
| Navab (2) | 2002 | apoE -/- mice | maintained on chow diet | female | 17 | D-4F | oral | 2.0 mg/mL | Daily/6 weeks | aortic root |
| Nayyar (1) | 2012 | apoE -/- mice | Normal died | female | 26 | 4F | ip | 100 μg/100 μl in saline | Daily/16 weeks | aortic sinus, En face percent lesion area |
| study | year | Animal | diet | Gender | N | Intervention | Route of administration | Dose | Duration | The artery used for lesion assessment |
| Nayyar (2) | 2012 | apoE -/- mice | Normal died | female | 26 | [K 4,15 >R]4F | ip | 100 μg/100 μl in saline | Daily/16 weeks | aortic sinus, En face percent lesion area |
| Nayyar (3) | 2012 | apoE -/- mice | Normal died | female | 26 | [K 9,13 >R]4F | ip | 100 μg/100 μl in saline | Daily/16 weeks | aortic sinus, En face percent lesion area |
| Ou (1) | 2012 | LDLR -/- mice | Western diet | male | 12 | D-4F | ip | (1 mg/kg | Daily/10 weeks | aorta |
| Ou (2) | 2012 | LDLR -/- mice | Western diet | male | 12 | D-4F | ip | (1 mg/kg | Daily/10 weeks | aorta |
| Qin | 2012 | apoE -/- mice | chow diet | female | 27 | Rev-D4F | oral | .4 mg/mL, equivalent to 1.6 mg/d | Daily/6 weeks | aortic root （aortic sinus sections ） |
| Tangirala | 1999 | LDLR -/- mice | Western diet | female | 23 | AdhapoA-I | Gene Transfection | / | / | aorta/aortic root |
| Wool | 2011 | apoE -/- mice | standard laboratory chow diet | female | 16 | 4F | ip | 50 μg 4F | Every other day/  4 weeks | innominate artery/ aorta/aortic root |
| Ying | 2003 | apoE -/- mice | high-fat diet | male | 12 | L-4F | ip | 1 mg/kg | Daily/16 weeks | aorta |
| Zhang (1) | 2010 | apoE -/- mice | fed a high fat, high-cholesterol | male | 11 | apoA-I(N74C) | iv | 40 mg/kg | Every 10 days/  30 days | carotid arteries |
| Zhang (2) | 2010 | apoE -/- mice | fed a high fat, high-cholesterol | male | 11 | rHDLwt | iv | 40 mg/kg | Every 10 days/30 days | carotid arteries |
| Zhang (3) | 2010 | apoE -/- mice | fed a high fat, high-cholesterol | male | 11 | rHDL Milano | iv | 40 mg/kg | Every 10 days/30 days | carotid arteries |
| Suematsu (1) | 2019 | apoE -/- mice | high-fat diet | male | 12 | FAMP | ip | 50 mg/kg | 2 times per week/16 weeks | aorta |
| study | year | Animal | diet | Gender | N | Intervention | Route of administration | Dose | Duration | The artery used for lesion assessment |
| Suematsu (2) | 2019 | apoE -/- mice | high-fat diet | male | 12 | i-FAMP-D1 | ip | 50 mg/kg | 2 times per week/16 weeks | aorta |
| Graversen | 2008 | LDLR -/- mice | hypercholesterolemic Western type diet | male | 26 | Trimeric apoA-I | ip | 100 mg/kg | twice weekly  /24 days | aortic roots |

apoE -/-: apolipoproteinE deficient, LDLR -/-: low density lipoprotein receptor deficient, Iv: intravenous injection, ip: intraperitoneal injection, ih: subcutaneously injection.

| Outcome or subgroup title | No. of studies | No. of participants | Effect size |
| --- | --- | --- | --- |
| 1.Final percent leisure area (%) | 23 | 269 | -1.75(-2.21, -1.29) |
| By gender |  |  |  |
| 1.1 male | 17 | 193 | -1.70(-2.26, -1.15) |
| 1.2 female | 6 | 76 | -1.94(-2.86, -1.02) |
| By genetic type |  |  |  |
| 1.3 LDL-/- mice | 7 | 82 | -1.75(-2.55, -0.96) |
| 1.4 apoE -/- mice | 16 | 187 | -1.77(-2.35, -1.19) |
| 2.Final leisure area(mm2) | 14 | 250 | -0.78(-1.18, -0.38) |
| By gender |  |  |  |
| 1.1 male | 3 | 52 | -0.28(-0.83,0.26) |
| 1.2 female | 7 | 130 | -1.21(-1.68, -0.74) |
| 1.3 not mentioned | 4 | 68 | -0.38(-1.26,0.51) |
| By genetic type |  |  |  |
| 1.4 LDL-/- mice | 4 | 70 | -1.16(-2.41,0.08) |
| 1.5 apoE -/- mice | 10 | 180 | -0.71(-1.12, -0.31) |

**Supplementary table4:Subgroup analysis of animal trails**

Lower final percent lesion area was observed in HDL/apoA-1 subgroups based on LDL-/- mice, apoE -/- mice, male, female; Lower Final lesion area was observed in HDL/apoA-1 subgroups based on female and apoE -/- mice. apoE -/-: apolipoproteinE deficient, LDLR -/-: low density lipoprotein receptor deficient.

| study  **Supplementary table 5: assessment of animal trails’ Scientific Inquiry (stair list)** | year | Sample size calculation | Inclusion and exclusion criteria | Randomization | Allocation concealment | Reporting of animals excluded from analysis | Blinded assessment of outcome | Reporting potential conflicts of interest and study funding |
| --- | --- | --- | --- | --- | --- | --- | --- | --- |
| Amar | 2010 | no | no | no | no | no | Yes | No |
| Averill | 2014 | Yes | no | no | no | no | yes | yes |
| Bielicki | 2010 | no | no | no | no | no | no | no |
| Bodary | 2004 | no | no | no | no | no | no | no |
| Ditiatkovski | 2013 | no | no | no | no | No | no | no |
| Guo | 2018 | no | No | unclear | no | no | no | yes |
| Li | 2004 | no | no | no | no | no | no | no |
| Navab | 2002 | no | no | no | no | no | no | no |
| Nayyar | 2012 | no | no | unclear | no | no | no | no |
| Ou | 2012 | no | no | no | no | no | no | yes |
| Qin | 2012 | no | no | no | no | no | no | yes |
| Tangirala | 1999 | no | no | unclear | no | no | yes | no |
| Wool | 2011 | no | yes | no | no | yes | no | no |
| Ying | 2003 | no | no | unclear | no | no | no | yes |
| Zhang | 2010 | no | no | unclear | no | no | no | no |
| Suematsu | 2019 | no | no | no | no | no | no | yes |

Quality of all selected animal trail relevant papers was assessed by stair list: the result included 12 “yes”, 95 “no”, 5 “unclear”.
