# Supplementary material for: Efficacy and Safety of High-Density Lipoprotein/Apolipoprotein A1 Replacement Therapy in Humans and Mice With Atherosclerosis: A Systematic Review and Meta-Analysis
Source: Front Cardiovasc Med. 2021 Aug 2;8:700233. doi: 10.3389/fcvm.2021.700233 (PMC8377725; doi:10.3389/fcvm.2021.700233)
Supplement: Supplementary file 2 [file Data_Sheet_2.docx]

Efficacy and safety of HDL/apoA-1 replacement therapy in humans and mice with atherosclerosis: A systematic review and meta-analysis

Ayiguli Abudukeremu^1,#^, Canxia Huang^3,#^,Hongwei Li^1^, Runlu Sun^1,4^, Xiao Liu^1,4^, Xiaoying Wu^1,4^, Xiangkun Xie^1,4^, Jingjing Huang^1,4^, Jie Zhang^1,4^, Jinlan Bao^2^, Yuling Zhang^1,4,*^

^1^Cardiovascular Medicine Department, Sun Yat-sen Memorial Hospital, Sun Yat-sen University, No. 107, the West of Yanjiang Road West, Guangzhou, 510120, China

^2^Comprehensive Department, Sun Yat-sen Memorial Hospital, Sun Yat-sen University, No. 107, the West of Yanjiang Road West, Guangzhou, 510120, China

^3^Critical Care Medicine Department, Sun Yat-sen Memorial Hospital, Sun Yat-sen University, No. 107, the West of Yanjiang Road West, Guangzhou, 510120, China

^4^Guangdong Province Key Laboratory of Arrhythmia and Electrophysiology, Guangzhou, 510120, China

^#^Ayiguli Abudukeremu and Canxia Huang have equal contributions.

*Correspondence: Yuling Zhang, Department of Cardiology, Sun Yat-sen Memorial

Hospital, Sun Yat-sen University No. 107, the West of Yanjiang Road, Yuexiu District Guangzhou, China, 510120, Email: [zhyul@mail.sysu.edu.cn](mailto:zhyul@mail.sysu.edu.cn).





**Supplementary figure1:** The methodological quality of the human studies was assessed using Review Manager (RevMan) software(version 5.3.）





**Supplementary figure 2:** sensitivity analysis of the studies included in the meta-analysis human. percent atheroma volume (A) and total atheroma volume (B). SMD: standardized mean difference; CI: confidence interval. The results did not significantly change in the sensitivity analysis.

**Supplementary** **Figure3:** Forest plot of risk of headache(A), renal impairment (assessed by creatinine elevation) (B), hepatic impairment (assessed by aspartate or alanine aminotransferase or bilirubin elevation) (C), Nausea, vomiting, or abdominal pain(D), using a random-effects model. figure A, B, C, D: no significant difference in adverse effects is observed between experiment and control group. SMD: standardized mean difference; CI: confidence interval.


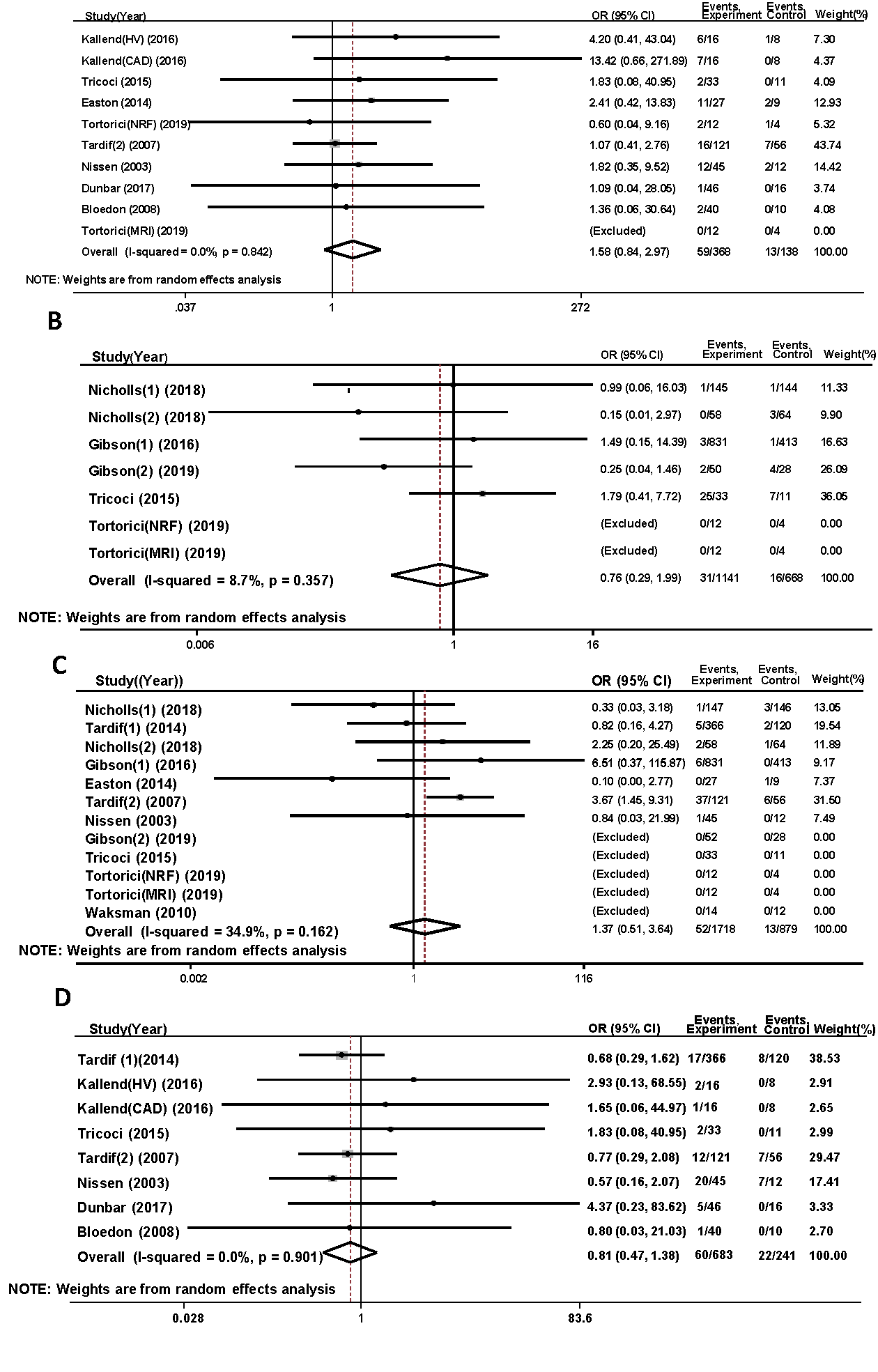





**Supplementary figure4:** sensitivity analysis of the studies included in the meta-analysis of adverse effect (A: headache, B: renal impairment, C: hepatic impairment, D: Nausea, vomiting, or abdominal pain). SMD: standardized mean difference; CI: confidence interval. The results did not significantly


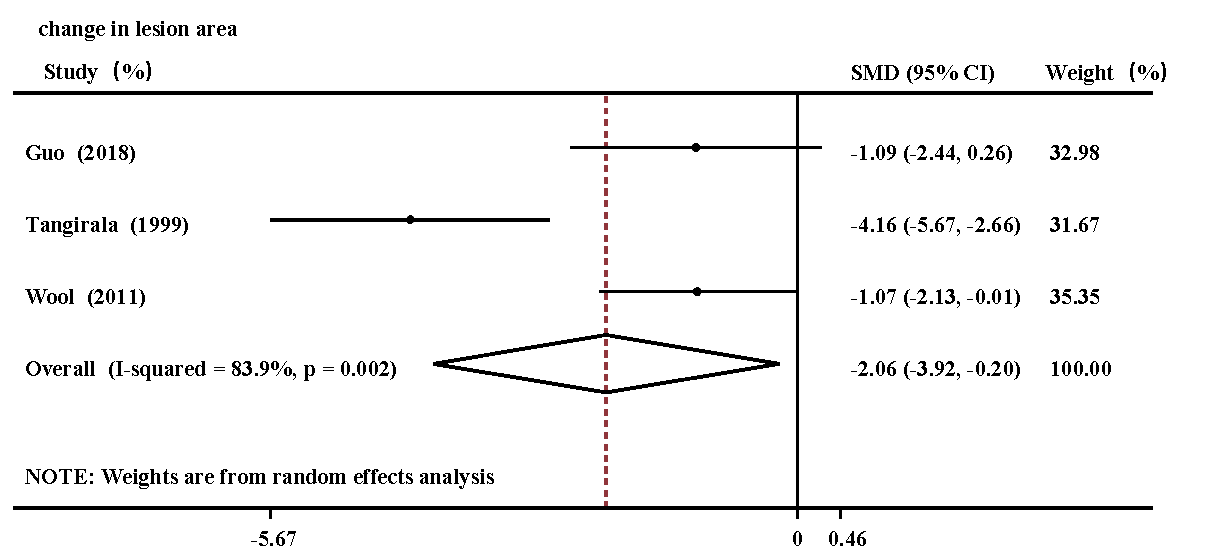


**Supplementary figure5:** Forest plot of the meta-analysis of the associations between change in lesion area in arteries and HDL/apoA-1 replacement therapy administration in mice using a random-effects model. SMD: standardized mean difference; CI: confidence interval. This result shows that HDL/apoA-1 replacement therapies significant change(decrement) in lesion area in mice with coronary atherosclerosis.


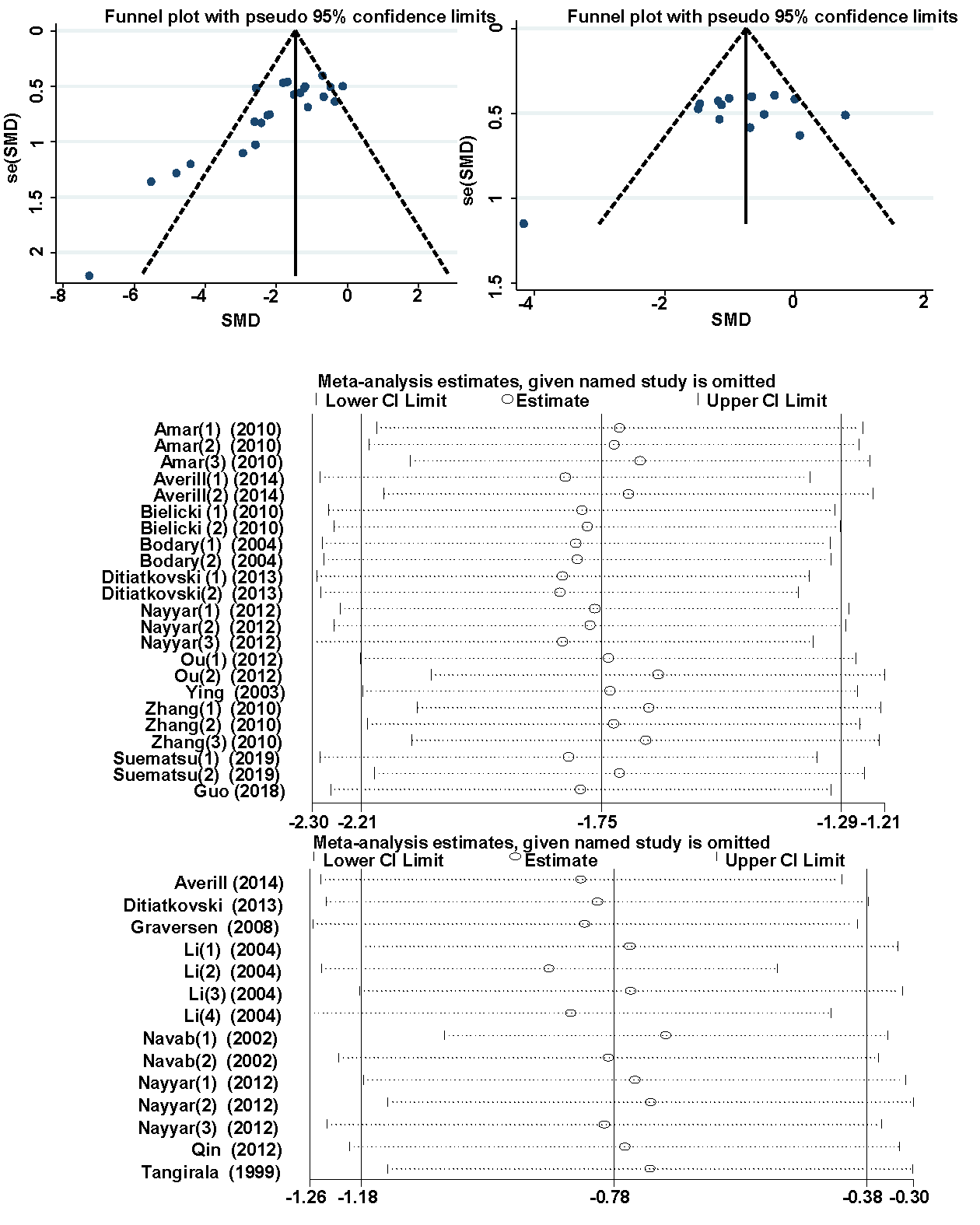


**Supplementary figure6:** Funnel plots and sensitivity analysis of the studies included in the meta-analysis of final percent lesion area (A, C) and final lesion area (B, D). SMD: standardized mean difference; CI: confidence interval. Both funnel plot show that one point (one study) located far from the other clustered points (other included studies) is responsible for the asymmetry in this meta-analysis. The results did not significantly change in the sensitivity analysis.
